# Treatment of Traumatic Intracranial Pseudoaneurysms: A Single-Center Experience

**DOI:** 10.3389/fneur.2021.690284

**Published:** 2021-06-25

**Authors:** Yingwu Shi, Yuan Gao, Yufei Liu, Wenxing Cui, Gaoyang Zhou, Liang Wang, Jia Yu, Tao Zhang, Yan Qu, Jianping Deng, Shunnan Ge

**Affiliations:** ^1^Department of Neurosurgery, Tangdu Hospital, Fourth Military Medical University, Xi'an, China; ^2^School of Aerospace Medicine, Fourth Military Medical University, Xi'an, China

**Keywords:** traumatic intracranial pseudoaneurysms, surgical treatment, endovascular treatment, parent artery, side branch artery

## Abstract

**Background and Purpose:** As a rare lesion secondary to brain trauma, traumatic intracranial aneurysms (TICAs) lead to high mortality and morbidity, and multiple treatment modalities have been applied for TICAs. All patients diagnosed with TICAs in our institution from 2010 to 2020 were included in the report, and their clinical features, treatment, and outcomes are described in detail. The purpose of this study is to illustrate the characteristic of different therapeutic methods of TICAs, and focus on the endovascular treatment.

**Methods:** A total of 20 patients were included in this study. The 3 patients who declined treatment all died. Five of the other 17 patients were treated surgically, including clipping, wrapping, and trapping with or without EC-IC high-flow bypass, with only 1 case of parent artery preservation. Twelve patients underwent endovascular treatment, including bare coil embolization (1 case), stent-assisted coiling (2 cases), balloon-assisted coils/Onyx glue embolization (1 case) and covered stents (8 cases), with only 1 case of parent artery sacrifice.

**Results:** 20 patients were included in the present study with 17 males, and the mean of age on 27 years (IQR: 22, 44 years). Eight patients presented with epistaxis, followed by 5 patients with coma, 3 patients with visual defects and 2 patients with CSF leakage. There were 18 TICAs located at the internal carotid artery (ICA); The other 2 TICAs located at pericallosal artery and A1 segment anterior cerebral artery (ACA). One case of diplopia occurred due to sacrifice of the ICA. Occlusion of the ophthalmic artery occurred in 3 patients after placement of a covered stent, with 1 patient suffering an irreversible vision decrease. None of the other patients who underwent the treatment have experienced an aggravation of their symptoms since the treatment; During the imaging follow-up, 1 case of recurrence and 1 case of endoleak occurred in this case series.

**Conclusions:** TICAs are associated with significant morbidity and mortality, and endovascular treatment has emerged as a valuable option, which may be promising to improve the clinical outcomes due to their advantages of preserving the parent artery if occlusion of the side branch artery can be avoided.

## Introduction

Traumatic intracranial aneurysms (TICAs) have been reported to be quite rare in large series, constituting <1% of all aneurysms (1, 2). TICAs may progressively grow and they have a high rate of rupture, which can be a fatal complication; early detection and subsequent aggressive treatment are generally needed (2, 3). Multiple modalities have been applied for treating TICAs, such as conventional open-surgical treatment and newly developed endovascular techniques (3–7). No single modality is indicated for all lesions, and therapeutic decisions rely on the location and shape of the aneurysms (3).

As the mainstream surgical treatment of intracranial aneurysms, clipping is an important method for treating TICAs (8, 9). However, since it can compromise the artery wall's patency, clipping is sometimes impossible, and wrapping and trapping may be the only choice (10–12). Wrapping has a risk of recurrence and rupture (12), and trapping should always be combined with complicated bypass procedures (10, 11). Therefore, surgical treatment of TICAs is a great challenge for neurosurgeons due to its pathological complexity and frequent close relationship with the skull base (6).

The development of endovascular techniques has made most complicated TICAs curable and dramatically reduces the risk of morbidity and mortality after surgery (3, 6). Bare or stent-assisted coil embolization and covered stent grafts have been applied for the treatment of TICAs and have shown promising efficacy and safety (3, 7, 13–15).

Here, we present a case series of TICAs in our center from 2010 to 2020 to illustrate the evolution of the therapeutic modalities for TICAs, and the characteristic of different therapeutic methods are discussed through a detailed description of the reported cases.

## Patients and Methods

### Patients

This study was approved by the Institutional Review Board of Tangdu Hospital, Fourth Military Medical University. We retrospectively reviewed all cases of TICAs from 2010 to 2020 according to the following criteria: (1) a clear history of head trauma or iatrogenic injury; (2) no history of intracranial aneurysms before the head injury; (3) no history of sudden headaches before the head injury; (4) no history of a loss of consciousness before the head injury; and (5) a cerebral angiography or computed tomography angiography (CTA) was performed soon after the head injury. As shown in Table 1, the patients' clinical data, including age, sex, presentations, etiology, TICA location and size, computed tomography (CT) findings, treatment, operating time, postoperative hospital stay, immediate results, complications and follow-up outcomes, were collected.

**Table 1:** Detailed clinical characteristics of the patients presenting with traumatic intracranial aneurysms.

	**Age (year)/sex**	**Incident**	**Aneurysm location**	**Aneurysm size**	**Presentations**	**CT findings**	**Skull base Frx**	**GSC score at angio**	**Treatment**	**Immediate result**	**Parent artery Preserved**	**Outcome**	**Complications**
1	12/F	MVA	R Pericallosal artery	5 × 4 mm	Right CN II damage, diabetes insipidus	ICH/C	Yes	15	Clipping	Aneurysm obliteration	Yes	Angio f/u: AEC, GOS score 5	None
2	22/M	MVA	R ophthalmic segment ICA	21.6 × 5.4 mm	Epistaxis, CSF rhinorrhea, right CN III damage	EDH, ICH/C	Yes	15	Covered stent (Jostent)	Aneurysm incomplete obliteration	Yes	CTA f/u: Recur, GOS score 4	None
3	22/M	MVA	L PcomA segment ICA	5 × 6 mm	Headache, dizzyness	SAH	No	15	Wrapping	Aneurysm obliteration	Yes	Lost of f/u	None
4	25/M	Fall	R ophthalmic segment ICA &CCF	6.4 × 8.7 mm	Bilateral visual defect, CSF otorrhea	ICH/C	Yes	15	Stent-assisted coiling	Aneurysm obliteration	Yes	Angio f/u: AEC, GOS score 5	None
5	23/M	Fall	R ophthalmic segment ICA	7.6 × 6.6 mm	Epistaxis	Cranial Frx	Yes	15	Covered stent (Willis)	Aneurysm obliteration	Yes	Angio f/u: AEC, GOS score 5	None
6	44/M	TA	L ophthalmic segment ICA	1.8 × 2.2 mm	Right CN VI damage	SAH	No	13	Stent-assisted coiling	Aneurysm obliteration#	Yes	Angio f/u: AEC, GOS score 4	None
7	60/F	Blunt head injury	R ophthalmic segment ICA	1.5 × 1.8 mm	Coma	ICH/C, SAH	No	9	No treatment	N/A	N/A	N/A; Dead	N/A
8	28/M	Blunt head injury	R ophthalmic segment ICA	6.5 × 7.4 mm	Epistaxis	SAH	No	15	Covered stent (Willis)	Small endoleak#	Yes	Angio f/u: Residual, GOS score 5	None
9	43/M	Iatrogenic	R petrous segment ICA	21 × 13 mm	Epistaxis	None	No	15	Covered stent (Willis)	Aneurysm obliteration	Yes	Angio f/u: AEC; Dead	None
10	22/M	TA	L PcomA ICA	19.9 × 18.6 mm	Coma	ICH/C, SAH	Yes	5	Trapping	Aneurysm obliteration	No	Angio f/u: AEC, GOS score 4	None
11	26/M	TA	R cavernous segment ICA	6.4 × 11.9 mm	Epistaxis	SDH	Yes	15	Balloon-assisted coils/Onyx embolization	Aneurysm obliteration	Yes	Angio f/u: AEC, GOS score 4	None
12	28/M	Fall	L ophthalmic segment ICA	7.6 × 8.2 mm	Epistaxis	Cranial Frx, pneumocephalus	Yes	15	Covered stent (Willis)	Aneurysm obliteration	Yes	Angio f/u: AEC, GOS score 5	None
13	29/M	Fall	R cavernous segment ICA &CCF	2.3 × 1.6 mm	L facial paralysis	ICH/C, Cranial Frx	Yes	15	Covered stent (Willis)	Aneurysm obliteration	Yes	Angio f/u: AEC, GOS score 5	None
14	19/M	TA	R PcomA segment ICA	9.8 × 8.5 mm	L hemiparesis	ICH/C, SDH	No	10T	Trapping & EC-IC high-flow bypass	Aneurysm obliteration	No	Angio f/u: AEC, GOS score 3	None
15	46/M	MVA	L PcomA segment ICA &CCF	10.6 × 3.9 mm	Conjunctival congestion	ICH/C, SAH	Yes	15	Coiling	Aneurysm obliteration	No (Balloon)	Angio f/u: AEC, GOS score 4	Diplopia
16	20/M	TA	R A1 segment ACA	12.6 × 19.7 mm	Coma, CSF rhinorrhea	ICH/C, SAH, IVH	Yes	4	No treatment	N/A	N/A	N/A; Dead	N/A
17	39/M	MVA	L choroidal segment ICA	2 × 2.2 mm	Regain consciousness from coma	Cranial Frx, SAH	Yes	15	Wrapping	Aneurysm obliteration	No	Angio f/u: AEC, GOS score 5	None
18	24/M	TA	R cavernous segment ICA	2.6 × 4.9 mm	Epistaxis	Cranial Frx	Yes	15	Covered stent (Willis)	Aneurysm obliteration#	Yes	Angio f/u: AEC, GOS score 5	Vision decrease
19	47/M	Fall	R cavernous segment ICA	5 × 5.7 mm	R CN II&III damage	SDH, EDH, SAH	Yes	15	Covered stent (Willis)	Aneurysm obliteration#	Yes	Angio f/u: AEC, GOS score 5	None
20	47/F	Iatrogenic	L PcomA	7.2 × 5.5 mm	Coma, epistaxis, epilepsy	SAH	No	4	No treatment	N/A	N/A	NA; Dead	N/A

*#Indicated the occlusion of the ophthalmic artery. AEC, aneurysm excluded from circulation; Angio, angiography; CCF, carotid-cavernous fistula; CN, cranial nerve; CT, computed tomography; CTA, computed tomographic angiography; EC-IC, extracranial-intracranial; EDH, epidural hematoma; Frx, fracture; f/u, follow-up; GCS, Glasgow Coma Scale; GOS, Glasgow Outcome Scale; ICA, internal carotid artery; ICH/C, intracranial hemorrhage/contusion; L, left; MVA, motor vehicle accident; PcomA, posterior communicating artery; R, right; SAH, subarachnoid hemorrhage; SDH, subdural hemorrhage; TA, traffic accident*.

### Clinical and Imaging Follow-Up

Immediate digital subtraction angiography (DSA) after the surgical procedures was performed to evaluate the complete obliteration of the aneurysms and the residual tissue. The degree of occlusion following treatment was stratified according to the Raymond–Roy Occlusion Classification (RROC). RROC I aneurysms are defined as complete obliteration of aneurysms, RROC II or III aneurysms are defined as residual aneurysm (16). Clinical outcomes were assessed throughout hospitalization and the patients were followed up until one year after discharge by telephone interview. DSA or CTA was reperformed one year after the initial surgery for imaging follow-up. Head CT examinations were performed at 3, 6, and 12 months postoperatively, along with re-examination of the patient's physical condition, and the clinical follow-up outcome was defined by the patient's Glasgow Outcome Scale (GOS) score.

### Statistics Analysis

All statistical analyses were performed using IBM SPSS Statistics 20.0 (IBM). Continuous variables are presented as the median (interquartile range) if the distributions were skewed or as the mean ± standard deviation (SD). Two independent groups were compared using Mann-Whitney test. *P* < 0.05 was considered statistically significant.

## Results

### Summary of the Cases

During the 10-year period from January 2010 to March 2020, 20 patients with TICAs were treated and included in the present study, accounting for 0.21% of all aneurysm cases (9936 cases) diagnosed in our center during the corresponding period. As shown in Table 1, traffic accidents (TAs) were the most common causes (6 cases) of TICAs, followed by motor vehicle accidents (MVAs) (5 cases) and falls (5 cases). 2 cases were due to direct blunt head injuries. In this case series, there were 2 iatrogenic TICAs, with 2 located at the right A1 segment of the ACA and the initial segment of the PcomA secondary to endoscopic surgical treatment for pituitary adenomas and the other located at the petrous segment of the ICA secondary to radiotherapy for sphenoidal malignant tumors. The male to female ratio was 17:3, and the mean of age on 27 years (IQR: 22, 44 years; ranged: 12–60 years), with an average age of 31.3 ± 12.6 yrs. Operation time in endovascular treatment was shorter than surgical treatment (2.35 ± 1.37 vs. 5.39 ± 3.97, *U* = 2.444, *P* = 0.015); there was no difference in postoperative hospital stay (13.08 ± 5.93 vs. 18.20 ± 3.96, *U* = 1.749, *P* = 0.082) (Supplementary Figure 1).

#### Presentations

Among all cases (Table 1), 8 patients (8/20 = 40.0%) presented with epistaxis, which was the most common problem, followed by CSF leakage (2 CSF rhinorrhea and 1 CSF otorrhea, 3/20 = 15.0%) and visual defects (3/20 = 15.0%). Nine patients were still in a coma when their TICAs were diagnosed after the trauma, with one presenting with CSF rhinorrhea, one presenting with right paralysis, 1 presenting with diabetes insipidus, 1 presenting with left facial spasm and 1 presenting with left conjunctival congestion (due to the comorbidity of the CCF) on physical examination (PE). One patient presented with right oculomotor nerve damage, manifesting diplopia on physical examination. One patient complained of headache and dizziness after a head trauma, and an aneurysm was diagnosed by DSA.

Except for the 2 iatrogenic TICAs, 16 TICAs were located at the ICA, with 7 at the ophthalmic segment, 4 at the cavernous segment, 4 at the PcomA segment and 1 at the choroidal segment. Most of the ICA TCA cases were related to skull fractures or skull base fractures, with 3 cases coexisting with CCF. In addition, there was one TICA located in the pericallosal segment of the ACA and the other TICA was located at the A1 segment of the ACA. CT manifestations included subarachnoid hemorrhage (SAH), intracranial hemorrhage/contusion (ICH/C), epidural hemorrhage (EDH), subdural hemorrhage (SDH), intraventricular hemorrhage (IVH) and pneumocephalus.

#### Treatments

Among all of the cases (Table 1), 5 patients underwent neurosurgical treatment, with 1 receiving clipping, 2 receiving wrapping, 1 receiving trapping and 1 receiving trapping combined with EC-IC high-flow bypass procedures. In addition, 12 patients underwent endovascular treatment. Among these cases, most (8/12 = 66.7%) patients received covered stent placement, and the ophthalmic artery (OphA) was obstructed in 3 patients; although immediate angiography indicated that collateral flow from the external cranial artery (ECA) to the retina could be observed for all 3 patients, an irreversible vision decrease occurred in 1 patient. Additionally, 2 patients were treated by stent-assisted coiling, 1 patient was treated by bare coil embolization, and 1 received a balloon-assisted coil and Onyx glue embolization. The remaining three patients refused any treatment after the diagnosis of TICA.

#### Outcomes

Clinical follow-up data were collected from 20 patients, with 1 patient lost to follow-up. The 3 patients who did not undergo any treatment after the diagnosis were all confirmed to have died. All of the other 16 patients did not have any aggravation of their symptoms after the treatment, with GOS scores of 5 for 10 patients, 4 for 5 patients and 3 for 1 patient, with amean GOS score of 4.5. Follow-up imaging showed that there was 1 case of aneurysm recurrence and 1 case of a residual aneurysm due to a small endoleak during the initial therapy.

## Illustrative Cases

**Figure 1:**
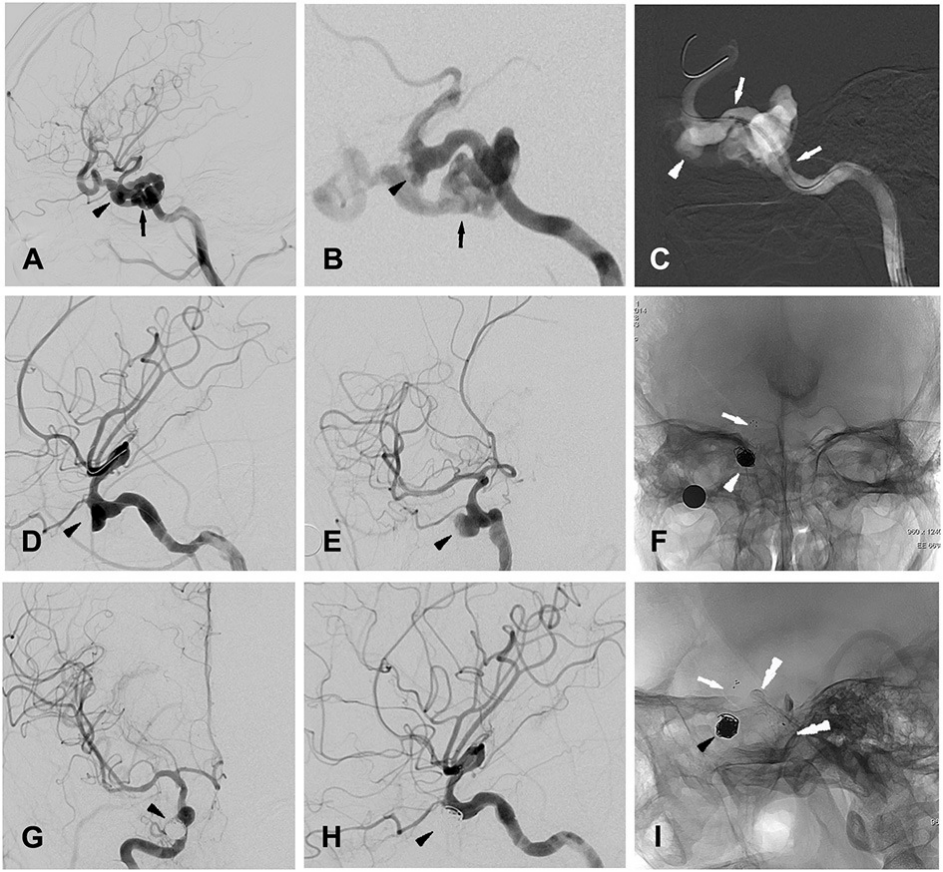
Patient 5 **(A,B)** (magnified), lateral **(A)** and oblique **(B)** views of right ICA angiogram showing the traumatic carotid-cavernous fistula (CCF, indicated by black arrow) as well as one pseudoaneurysm (indicated by black arrow head) located at cavernous segment of ICA. **(C)** Intra-procedural road map during stent graft (white arrows) positioning for occluding the CCF, with white arrow head indicating the pseudoaneurysm. **(D,E)** Oblique views of right ICA angiogram showing the complete occlusion of the CCF and the remained pseudoaneurysm (indicated by black arrow head). **(F)** Post-treatment radioscopic anteroposterior view of the placed coils (white arrow head) and stent (white arrow) for the stent-assisted coiling procedures. **(G,H)** Anteroposterior **(G)** and lateral **(H)** views of a right ICA angiogram showing the completely occluded traumatic pseudoaneurysm (black arrow head). **(I)** post-treatment radioscopic lateral view of the placed coils (black arrow head) and the stent (white arrow) for stent-assisted coiling, as well as the covered- stent (black double-arrow heads) for treating CCF.

Patient 5 a 25-year-old man was admitted to a hospital with loss of consciousness and bleeding from the external acoustic canal and the oral and nasal cavity after a 5-meter fall. Cranial CT confirmed bilateral frontal, right temporal and left occipital intracerebral contusions and multiple skull base fractures. The patient was transferred to our hospital, and the DSA examination confirmed a carotid-cavernous fistula (CCF) at the right ICA. A pseudoaneurysm 6.4 × 8.7 mm was also identified at the cavernous segment of the right ICA (Figures 1A–C).

The procedure was performed under general anesthesia after whole body heparinization. A 6-French guide catheter was selectively inserted into the internal carotid artery via the femoral artery. Using the methods described for patient 13, a Willis covered stent (4 × 16 mm, MicroPort) was first placed over the orifice of the fistula (Figures 1A–C,I), and immediate angiography confirmed correct placement of the stent, intact blood flow of the ICA and satisfactory occlusion of the fistula (Figures 1D,E). Then, with the guidance of a digital road map, a stent microcatheter (Rebar^TM^-027, ev3) was advanced to the parent artery of the pseudoaneurysm. With the aneurysm embolization microcatheter (Echelon^TM^-10, ev3) carefully placed into the aneurysm cavity with the guidance of a 0.014-in microguidewire (Transend^TM^-300, Boston Scientific), the first coil (8 × 30 mm, Jasper) was released into the aneurysm. Then, a stent (SOLITAIRE^TM^ AB-6-20, ev3) was positioned with a microcatheter and released to fully cover the aneurysm neck (Figures 1F,I). After that, further aneurysm occlusion with detachable coils was then performed using standard procedures. Immediate angiography confirmed the complete embolization of the pseudoaneurysm (Figures 1G,H), and after the procedure, the patient was given heparin for 2 days followed by oral administration of aspirin (100 mg/day) and clopidogrel (75 mg/day) for 3 months.

**Figure 2:**
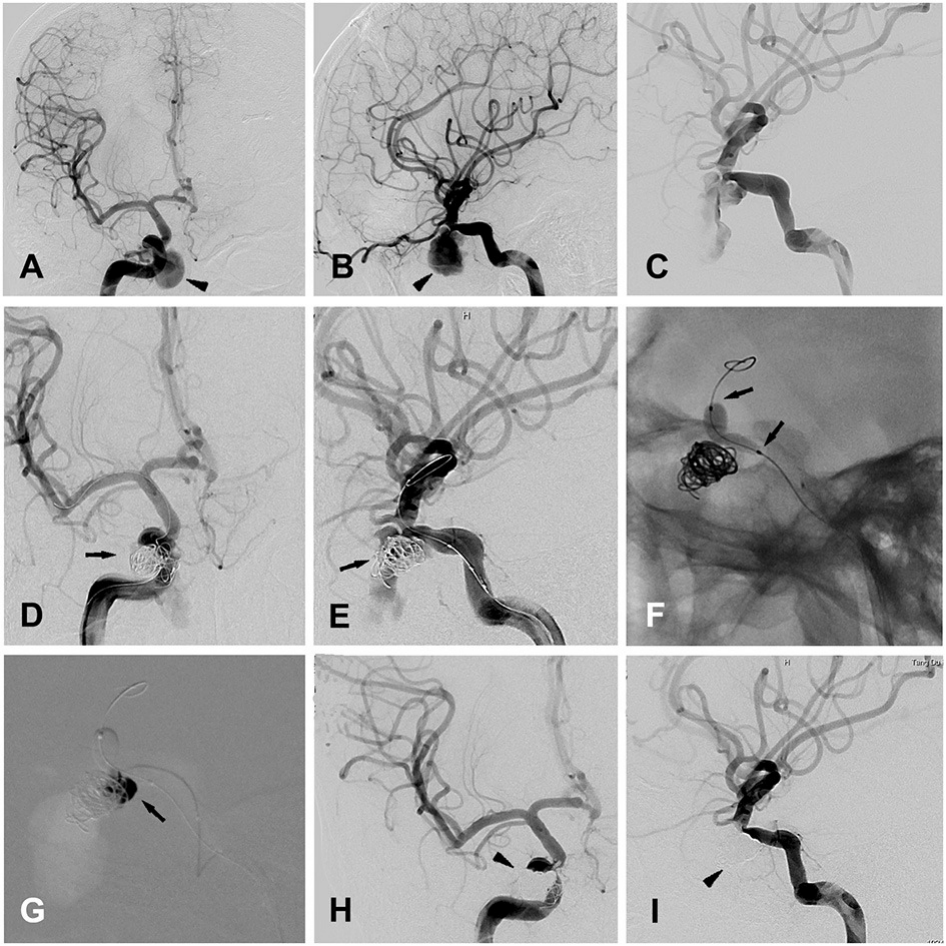
Patient 12 **(A,B)** anteroposterior **(A)** and lateral **(B)** views of the right ICA angiogram for initial diagnosis, showing a traumatic cavernous pseudoaneurysm (black arrow head). **(C)** The second time of right ICA angiogram before the embolization, showing the formation of the thrombus in the false cavity of the pseudoaneurysm. **(D,E)** Anteroposterior **(A)** and oblique **(B)** views of the right ICA angiogram, showing the placement of endovascular coils in the pseudoaneurysm (black arrows), with the embolization microcatheter directing toward the pseudoaneurysm's cavity. **(F)** Balloon inflation (black arrows) was accomplished with the saline/contrast mixture. **(G)** Intraprocedural road map during the injection of the onyx (black arrow) into the pseudoaneurysm's cavity, after the complete cover of the pseudoaneurysm's ostium by the inflated balloon. **(H,I)** Anteroposterior **(A)** and oblique **(B)** views of the right ICA angiogram, showing the traumatic pseudoaneurysm completely occluded by the coils and onyx embolization (black arrow heads).

Patient 12 a 26-year-old man was admitted to a hospital in a coma after a traffic accident. The patient immediately underwent decompressive craniectomy and evacuation of intracranial hematomas. A cranial CT scan confirmed right frontal acute subdural hemorrhage, as well as multiple cranial and skull base fractures. The patient then recovered, but 1 month later, he was admitted to our hospital due to repeated epistaxis, and a pseudoaneurysm 6.4 × 11.9 mm located at the cavernous sinus segment of the right ICA was found by initial DSA (Figures 2A,B). Three days later, a second DSA performed before embolization showed the formation of a thrombus in the false cavity of the pseudoaneurysm (Figure 2C).

The procedure was performed under general anesthesia after whole body heparinization. An 8-French guide catheter was selectively inserted into the left ICA via the femoral artery. Navigated over the microguide wire, a Hyperform balloon was first placed in the cavernous segment of the right ICA, bridging the ostium of the pseudoaneurysm under simultaneous biplanar road mapping. Then, an aneurysm embolization microcatheter (Echelon^TM^-10, ev3) was carefully placed into the aneurysm cavity with the guidance of a 0.014-in microguidewire (Traxcess 14EX), and aneurysm occlusion with detachable coils was performed using standard procedures (Figures 2D,E). Immediate angiography showed a significant reduction in blood flow in the cavity of the pseudoaneurysm (Figures 2D,E). Then, balloon inflation was accomplished with a 50:50 mixture of saline/contrast, and repeated angiography confirmed the correct placement of the balloon to completely cover the ostium of the pseudoaneurysm (Figure 2F). After that, 2.5 ml of Onyx glue (Onyx^TM^ 18) was injected into the cavity of the pseudoaneurysm via the embolization microcatheter (Figure 2G), and complete embolization of the pseudoaneurysm was confirmed by final angiography (Figures 2H,I). Finally, the balloon was deflated and retracted along with the microguidewire/microcatheter.

**Figure 3:**
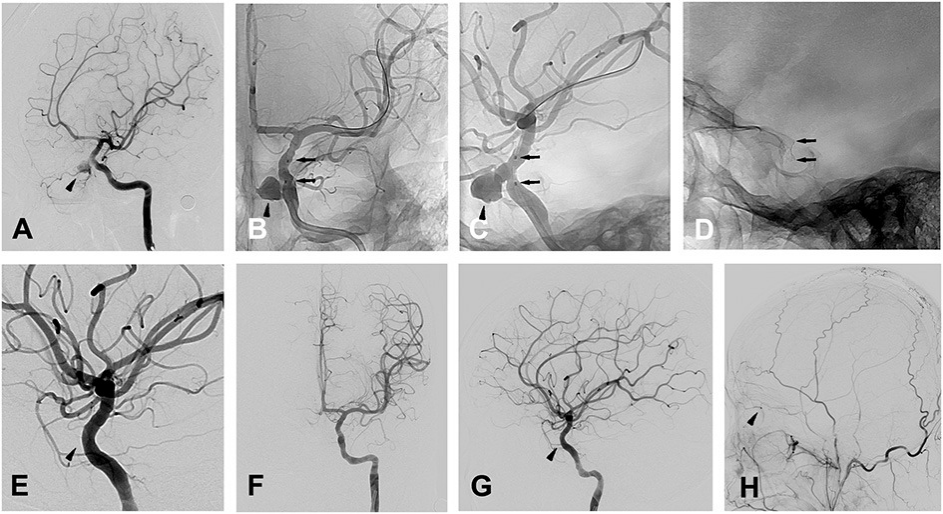
Patient 13 **(A)** oblique views of a left ICA angiogram showing the traumatic OphA aneurysm. **(B,C)** radioscopic anteroposterior **(B)** and oblique **(C)** views of the implanted stent during the left ICA angiogram. Black arrow heads indicate the pseudoaneurysm close to the ophthalamic artery in **(A–C)**, black arrows indicate the stent in **(B–G)**, oblique **(E)**, anteroposterior **(F)** and lateral **(G)** views of a left ICA angiogram showing the complete occlusion of the pseudoaneurysm, with black arrow head indicating the absence of the pseudoaneurysm as well as ophthalamic artery. **(H)** Lateral **(G)** view of a left ECA angiogram showing the presence of the collateral flow from ECA to OphA (black arrow head).

Patient 13 a 28-year-old man was admitted to a hospital for transient loss of consciousness after falling 6 meters and landing on his face. Cranial CT showed cranial fractures and pneumocephalus. Immediate severe epistaxis was stopped by nasal tamponade, and the patient rapidly recovered. One month later, the patient was admitted to our department because of the recurrence of severe epistaxis, and DSA confirmed a pseudoaneurysm 7.6 mm × 8.2 mm located at the OphA (ophthalmic artery) segment of the left ICA (Figures 3A–C).

Before the procedure, the patient took aspirin (100 mg/d) and clopidogrel (75 mg/d) for 3 consecutive days. Under general anesthesia, the procedure was performed after whole body heparinization. Then, a 6-French guiding catheter was inserted via the femoral artery and positioned in the left cervical ICA. With a tapered 0.014-in microguidewire (Transend^TM^-300, Boston Scientific) navigated into a distal branch of the middle cerebral artery, a Willis covered stent (4 × 10 mm, MicroPort) was navigated over the microguidewire until it bridged the ostium of the pseudoaneurysm with the ostium in the center of the stent under roadmap guidance (Figures 3C,D). Then, the stent was deployed with a balloon expanded by 5 atm pressure lasting for 5 s, and multiple control angiograms were performed immediately after balloon de?ation to confirm the correct placement of the stent and satisfactory exclusion of the pseudoaneurysm (Figures 3E,F). For the left OphA, which was occluded after stent placement (Figures 3E,G), additional angiography of the left ECA (external cranial artery) was conducted and collateral flow from the ECA to the retina was observed (Figure 3H) and the patient manifested no change in vision postoperatively. Heparin was given for at least 48 h after the procedure, and then the patient took aspirin (100 mg/d) and clopidogrel (75 mg/d) for 3 months to prevent thrombosis and in-stent stenosis.

## Discussion

TICAs have been reported to be rare lesions caused by blunt or penetrating trauma, constituting 0.15–0.4% of intracranial aneurysms (17). Reviewing 9,936 cases of intracranial aneurysms in our center from 2010 to 2020, 20 cases of TICAs were identified with an incidence of 0.21%, which was similar to previous reports (2). Traffic accidents, motor vehicle accidents and falls were the primary causes of TICAs in the presented cases, indicating that great external forces during head trauma are requisites for the development of TICAs.

TICAs have been reported to have a high mortality (1, 2); actually, the primary brain trauma itself is often lethal as well. For 4 cases presenting for treatment to our department, 2 were in a coma with GSCs of 8 and 4, and the families decided against treatment, even though TICAs were detected. Most of the described cases were transferred to our center from external hospitals and were in a good condition when they arrived at our hospital. Most of their TICAs were identified by CTA or DSA among patients who were recovering from trauma with TICA-related symptoms. We speculated that some patients might have died in external hospitals, possibly before their TICAs were diagnosed or re-ruptured. TICAs are potentially fatal lesions needing early diagnosis and early treatment.

Among the presenting symptoms, epistaxis was the most frequent in our series, which is similar to other studies (18, 19). In addition, CSF rhinorrhea, CSF otorrhea, diabetes insipidus, and damage to the cranial nerve have also been reported before (20). All of these symptoms prompted us to conduct angiographic scans for TICAs in brain trauma patients. CTA has been suggested as the perfect tool to scan intracranial aneurysms (2), including TICAs, which can be seen in the present series. However, some studies have suggested that for patients at a high risk of TICAs who have negative findings on CTA, proceeding with DSA for confirmation should be a necessary option as the “gold standard”(3).

In the present study, nearly all of the TICAs (17/20 = 85%) were located at the ICA. It has been well-established that direct injury to the vessel or stretching of the vessel by adjacent forces is the main mechanism causing TICAs (20). The close anatomical relationship between the ICA and the cranial skull base causes the ICA to be the site where TICAs mostly occur, especially when cranial base fractures occur after head trauma (6), which was prevalent in our series.

There was 1 case of TICA located at the pericallosal artery, which has also been reported to be another common site of TICAs (20), occurring due to laceration of the distal pericallosal artery branch under the sharp edge of the falx cerebri (21). In addition, our series reported 2 cases of iatrogenic TICAs, with 1 located at the PcomA after transnasal endoscopic surgery and 1 located at the petrous ICA after radiotherapy. These types of TICAs are caused by direct injuries of vessels by surgical procedures or radiation exposure; thus, their location was atypical.

In the present study, the size of the aneurysm varied dramatically, with diameters ranging from 1.5 to 21.6 mm, which was similar to previous reports (4, 14). In fact, pseudoaneurysms are the cavity of an encapsulated hematoma communicating with the arterial lumen (3); therefore, the size and shape of the pseudoaneurysm varies dramatically and can even change rapidly, as shown by patient No. 12, due to the formation of a thrombus in the cavity.

Because of the fragile wall and the broad or obscure neck, direct surgical repair of TICAs is challenging (2, 8). Conventional surgical procedures, including clipping, wrapping, and trapping with or without EC-IC bypassing, have been used for the management of TICAs (8). In our series, only one iatrogenic pseudoaneurysm located at the ACA and one traumatic pseudoaneurysm located at the pericallosal artery achieved satisfactory clipping, while for most cases, clipping was impossible due to the lack of an aneurysm neck (21). In addition, difficulty in accessibility, e.g., lesions located at the ICA, can also make surgical clipping impossible; thus, wrapping and trapping was the only choice before the development of endovascular procedures (6). Some authors have used cervical ICA ligation and aneurysm trapping or excision for treating TICAs (22–24), which do not induce terrible complications when collateral flows are present and preserve good functionality, and one case located at PcomA treated by trapping in our series conforms to this condition.

However, when insufficiency of the collateral flows occurs, which can involve intolerance of the BOT, EC-IC high-flow bypass is needed prior to aneurysm trapping to revascularize the ICA, which has been previously reported by several studies (10, 11).

Wrapping, another alternative technique utilized when an intracranial aneurysm is not amenable to direct clipping or the neck of the aneurysm cannot be clipped completely, was used for treating complex TICAs before the development of endovascular modalities (8). Deshmuck et al. reported a large series of intracranial aneurysms treated by the wrapping technique. Three cases of traumatic pseudoaneurysms located at the ophthal, PCA and Pcom were wrapped by cotton, with all demonstrating a stable angiographic outcome (25). However, the relatively high risk of re-bleeding with a reported incidence of 11.9–27.7% (26), as well as granuloma formation and cerebrovascular complications, limited this method's further application. In our series, 2 cases of ICA TICAs treated by wrapping all achieved a good recovery, and no re-bleeding occurred within the follow-up period; however, whether other cerebrovascular complications occurred was unknown.

Although the cases treated by open surgery in our series obtained a good recovery, the procedure is still challenging due to difficulties in accurate positioning, a greater possibility of premature rupture and limited visual fields during surgical dissection in the acute stage (12), and occasionally some lesions are inaccessible for direct surgical treatment, e.g., complex ICA TICAs. Notably, surgical treatment has been indicated to be associated with significant morbidity and a reported mortality of 18–29% (3, 22).

The development of endovascular techniques provides valid alternatives for treating TICAs. Due to the advantages of minimal manipulation of the adjacent brain tissue and vessels, a shorter operative time, and direct access to lesions by endovascular approaches, most cases treated after 2013 in our center were treated with new techniques, including bare coil embolization, stent-assisted coiling, balloon-assisted coiling, and covered stents. Bare coil embolization is well-known to be strictly applicable for only aneurysms with small necks, while the broad or obscure neck of TICAs suggests coil embolization always requires balloon or stent-assisted coiling.

As one of the earliest endovascular solutions for the treatment of wide-necked aneurysms, balloon-assisted coiling (BAC) was first used by Moret et al. for treating 52 aneurysms, with a complete occlusion rate of 77% (27). BAC, which places a removable balloon adjacent to the aneurysm, can prevent coil herniation into the parent vessel and help the coil assume the 3D shape of the aneurysm and thus achieve denser packing (28). We used these techniques for treating TICAs, applying coil and glue embolization to these false aneurysms.

In addition, stent-assisted coiling has advantages similar to BAC while treating wide-necked aneurysms. It has been reported that stent-assisted coiling achieved better complete occlusion rates of aneurysms at 6 months or later after the procedure compared to BAC (28), and stent-assisted coiling has been used for treating TICAs by several authors. In our series, 2 patients received stent-assisted coiling, and both achieved complete occlusion of the TICA. In addition, patency of the parent vessel was preserved in these two cases. Based on our results, despite the lack of a solid aneurysm wall, TICAs can be successfully treated by coil embolization with no severe complications.

However, coil embolization has several problems that cannot be ignored, including recurrence, incomplete obliteration, and obvious mass effects (29). Covered stents have emerged as a promising therapeutic option for treating complex cerebrovascular diseases in recent years (3, 15). Their working mechanism involves immediate exclusion of aneurysms from the intracranial circulation while preserving the patency of the parent artery. Covered stents have been applied more frequently in recent years in the management of complex aneurysms, such as BBAs and TICAs (29).

Stent grafts were first reported to be used for treating life-threatening subclavian posttraumatic hemorrhage by Becker et al. (30). Subsequently, studies reported the use of an autologous vein-covered Palmaz stent to occlude traumatic pseudoaneurysms in cervical ICA and CCA (7, 31). Although these veins are less likely to develop thrombosis and infection, this technique is still inconvenient because the saphenous vein needs to be harvested to cover the stent, and severe (90%) asymptomatic stenosis owing to stent compression has been reported after the use of balloon expandable deployment (6). With the development of expendable PTFE-covered stents, more intracranial TICAs have been reported to be treated by covered stents (6). Although the deployment of stents in intracranial ICAs is challenging due to the tortuous access, Redekop and colleagues applied a Jostent covered stent and a balloon-expandable eFTFE covered stent to completely exclude traumatic pseudoaneurysms located at petrocavernous and petrous ICAs, with a patent IVA (7). Celil et al. reported a case of successful occlusion of the TICA located in the cavernous ICA by a Jostent covered stent (32). Yi et al. reported 2 patients with cavernous carotid pseudoaneurysms treated with a Jostent covered stent, showing promising results in that all cases obtained complete occlusion with no recanalization during the follow-up period (33). Cohen et al. reported a series of 13 patients with one ICA cavernous lesion who underwent Jostent cover-stent placement, demonstrating full exclusion of the aneurysms and perfect ICA patency (3).

Subsequently, another type of balloon-expandable eFTFE covered stent, the Willis covered stent, has also been widely used for treating extra and intracranial traumatic pseudoaneurysms. Chen reported a large series of TICA cases that manifested repeated epistaxis with lesions all located in the siphon and cavernous sinus segment of the ICA. The Willis covered stent was used to successfully treat 11 patients with no repeated epistaxis or neurological deficits (34). Wang et al. reported treatment of 14 delayed pseudoaneurysms with a Willis covered stent in 13 patients, with an immediate complete exclusion rate of 71.4% and a late complete exclusion rate of 92.9%. The patency of the ICA was maintained for all patients, the clinical follow-up demonstrated a full recovery rate of 11/13, and no procedure-related complications or deaths occurred. Liu et al. reported that the application of a Willis covered stent for 3 pediatric TICA cases also showed satisfactory therapeutic effects (35).

In our series, one and seven cases were treated with a Jostent and a Willis covered stent, respectively, with nearly all aneurysms fully occluded and the ICA patent, except for one case of endoleak. Although some studies indicated that balloon-expandable covered stents were more prone to collapse, crushing, and occlusion than self-expandable stents, our cases did not experience any of these problems for either the Jostent or Willis covered stents, which may be due to the small sample size. Additionally, there are other modes of covered stents for treating TICAs, all suggesting covered stents to be the most promising approach to occluding TICAs while leaving the patent arteries intact (14).

Although covered stents are an attractive therapeutic strategy for TICA, there are still some limitations in their clinical application (14). First, they have a poor target arrival rate in passing the tortuous intracranial vasculature. In our series, the cases treated by covered stents involved several segments of the ICA, and for the juvenile patients, target arrival could be obtained because the ICA was not too tortuous.

Second, endoleak is still a frequent issue. In the present case series, one small endoleak occurred in patient 9, accounting for 12.5% of all cases when applying a covered stent, and a possible reason may be improper selection of the stent. Although the subsequent follow-up suggested a decrease in the endoleak, previous reports suggested that observation could be an advisable choice for managing this condition, which might offer a chance for spontaneous occlusion of endoleaks with minimal slow residual fill. However, the risk of an aneurysm rupture remains until the endoleak is completely occluded and the aneurysm lumen has completely thrombosed (14).

Finally, the placement of a covered stent induces the concern that important side branches or perforating arteries stemming from the covered artery segment would be closed. In our series, occlusion of the side branches was sometimes observed, e.g., the ophthalmic artery, but it may not result in a bad outcome due to good collateral flows, while sometimes the situation is different. As shown by patient 9, occlusion of the ophthalmic artery by a covered stent induced irreversible visual defects. Therefore, before the placement of the covered stent, the super-selective balloon occlusion test for the important side branches or perforating arteries should be performed before the deployment of the covered stent.

Based on the clinical outcomes of our case series, TICAs should obtain a satisfactory prognosis if timely diagnosed and properly treated. However, due to the small sample size and the lack of a randomized comparative design, we cannot determine which type of treatment modality was clearly better. This limitation is common in similar studies, mainly because TICA is a rare type of aneurysm. The other limitation of this study is CCF likely existed and ruptured in to the cavernous sinus due to trauma, because the aneurysms are not necessarily traumatic. In addition, longer follow-up was needed for our case series, which was crucial for finding and evaluating more complications, including recurrence, ICA occlusion, and in-stent stenosis. Therefore, long-term follow-up and large-scale RCTs with collaborations among multiple large institutions are needed in the future.

## Conclusion

An early angiographic diagnosis is crucial for a good outcome of patients suffering from TICAs. Endovascular modalities were applied in most cases in our present series, and nearly all obtained satisfactory short-term and long-term outcomes. The preservation of the branch arteries is still a significant challenge for the endovascular treatment of TICAs; thus, careful preoperative evaluation, such as super-selective balloon occlusion tests, are crucial, or irreversible complications may occur. Different techniques should be selected for specific cases according to the specific features of the lesions.

## Data Availability Statement

The raw data supporting the conclusions of this article will be made available by the authors, without undue reservation.

## Ethics Statement

The studies involving human participants were reviewed and approved by Tang Du Hospital of Fourth Military Medical University institutional review board. The patients/participants provided their written informed consent to participate in this study. Written informed consent was obtained from the individual(s) for the publication of any potentially identifiable images or data included in this article.

## Author Contributions

SG, JD, YS, YG, and YL contributed with study design, data collection, data analysis, interpretation of findings, and writing of the manuscript. WC, GZ, LW, and JY contributed with data collection and data analysis. TZ and YQ contributed with literature research. All authors contributed to the article and approved the submitted version.

## Conflict of Interest

The authors declare that the research was conducted in the absence of any commercial or financial relationships that could be construed as a potential conflict of interest.

## References

[1] Holmes B Harbaugh RE. Traumatic intracranial aneurysms: a contemporary review. J Trauma. (1993) 35:855–60. 10.1097/00005373-199312000-00009 8263982

[2] Dubey A Sung WS Chen YY Amato D Mujic A Waites P. Traumatic intracranial aneurysm: a brief review. J Clin Neurosci. (2008) 15:609–12. 10.1016/j.jocn.2007.11.006 18395452

[3] Cohen JE Gomori JM Segal R Spivak A Margolin E Sviri G. Results of endovascular treatment of traumatic intracranial aneurysms. Neurosurgery. (2008) 63:476–85; discussion 85–6. 10.1227/01.NEU.0000337169.55304.A0 18812959

[4] Bell RS Vo AH Roberts R Wanebo J Armonda RA. Wartime traumatic aneurysms: acute presentation, diagnosis, and multimodal treatment of 64 craniocervical arterial injuries. Neurosurgery. (2010) 66:66–79; discussion 10.1227/01.NEU.0000361285.50218.A8 20023539

[5] Niu Y Zhou S Tang J Miao H Zhu G Chen Z. Treatment of traumatic intracranial aneurysm: experiences at a single center. Clin Neurol Neurosurg. (2020) 189:105619. 10.1016/j.clineuro.2019.105619 31812032

[6] Spanos K Karathanos C Stamoulis K Giannoukas AD. Endovascular treatment of traumatic internal carotid artery pseudoaneurysm. Injury. (2016) 47:307–12. 10.1016/j.injury.2015.09.015 26453153

[7] Redekop G Marotta T Weill A. Treatment of traumatic aneurysms and arteriovenous fistulas of the skull base by using endovascular stents. J Neurosurg. (2001) 95:412–9. 10.3171/jns.2001.95.3.0412 11565861

[8] Jung SH Kim SH Kim TS Joo SP. Surgical treatment of traumatic intracranial aneurysms: experiences at a single center over 30 years. World Neurosurg. (2017) 98:243–50. 10.1016/j.wneu.2016.11.005 27836703

[9] Sui M Mei Q Sun K. Surgical treatment achieves better outcome in severe traumatic pericallosal aneurysm: case report and literature review. Int J Clin Exp Med. (2015) 8:1598–603. 25932088 PMC4402735

[10] Kankane VK Warade AG Misra BK. Extracranial-intracranial high-flow bypass for post-traumatic cavernous carotid pseudo-aneurysm presenting with epistaxis: case report. Neurol India. (2019) 67:485–90. 10.4103/0028-3886.257994 31085865

[11] Sun L Li M Zhang H Du J Ling F. Trapping with high-flow bypass for a traumatic giant pseudoaneurysm of the supraclinoid carotid artery in an adolescent: case report. Childs Nerv Syst. (2011) 27:681–4. 10.1007/s00381-011-1397-x 21279362

[12] He Y Wang L Ou Y Wang H Wang S Zhang P. Surgical treatment of traumatic distal anterior cerebral artery aneurysm: a report of nine cases from a single centre. Acta Neurochir. (2020) 162:523–9. 10.1007/s00701-019-04121-x 31802275

[13] Seth R Obuchowski AM Zoarski GH. Endovascular repair of traumatic cervical internal carotid artery injuries: a safe and effective treatment option. AJNR Am J Neuroradiol. (2013) 34:1219–26. 10.3174/ajnr.A3337 23221950 PMC7964571

[14] Liu Y Yang HF Xiong ZY Zheng J Liu CY Zhao HY. Efficacy and safety of willis covered stent for treatment of complex vascular diseases of the internal carotid artery. Ann Vasc Surg. (2019) 61:203–11. 10.1016/j.avsg.2019.05.027 31381999

[15] Maras D Lioupis C Magoufis G Tsamopoulos N Moulakakis K Andrikopoulos V. Covered stent-graft treatment of traumatic internal carotid artery pseudoaneurysms: a review. Cardiovasc Intervent Radiol. (2006) 29:958–68. 10.1007/s00270-005-0367-7 16897263

[16] Roy D Milot G Raymond J. Endovascular treatment of unruptured aneurysms. Stroke. (2001) 32:1998–2004. 10.1161/hs0901.095600 11546888

[17] Uzan M Cantasdemir M Seckin MS Hanci M Kocer N Sarioglu AC. Traumatic intracranial carotid tree aneurysms. Neurosurgery. (1998) 43:1314–20; discussion 20–2. 10.1227/00006123-199812000-00024 9848844

[18] Zhang C Xie X You C Mao B Wang C He M. Endovascular treatment of traumatic pseudoaneurysm presenting as intractable epistaxis. Korean J Radiol. (2010) 11:603–11. 10.3348/kjr.2010.11.6.603 21076585 PMC2974221

[19] Han MH Sung MW Chang KH Min YG Han DH Han MC. Traumatic pseudoaneurysm of the intracavernous ICA presenting with massive epistaxis: imaging diagnosis and endovascular treatment. Laryngoscope. (1994) 104(3 Pt 1):370–7. 10.1288/00005537-199403000-00021 8127196

[20] Larson PS Reisner A Morassutti DJ Abdulhadi B Harpring JE. Traumatic intracranial aneurysms. Neurosurg Focus. (2000) 8:e4. 10.3171/foc.2000.8.1.1829 16906700

[21] Van Rooij WJ Van Rooij SB. Endovascular treatment of traumatic pericallosal artery aneurysms. A case report. Interv Neuroradiol. (2013) 19:56–9. 10.1177/159101991301900108 23472724 PMC3601618

[22] Parkinson D West M. Traumatic intracranial aneurysms. J Neurosurg. (1980) 52:11–20. 10.3171/jns.1980.52.1.0011 7350269

[23] Enomoto H Shibata T Ito A Harada T. Traumatic aneurysm of the supraclinoid internal carotid artery: report of a case. Neurosurgery. (1984) 15:700–2. 10.1227/00006123-198411000-00011 6504285

[24] Jakobsson KE Carlsson C Elfverson J von Essen C. Traumatic aneurysms of cerebral arteries. A study of five cases. Acta Neurochir. (1984) 71:91–8. 10.1007/BF01401153 6731059

[25] Deshmukh VR Kakarla UK Figueiredo EG Zabramski JM Spetzler RF. Long-term clinical and angiographic follow-up of unclippable wrapped intracranial aneurysms. Neurosurgery. (2006) 58:434–42; discussion−42. 10.1227/01.NEU.0000199158.02619.99 16528182

[26] Perrini P Montemurro N Caniglia M Lazzarotti G Benedetto N. Wrapping of intracranial aneurysms: Single-center series and systematic review of the literature. Br J Neurosurg. (2015) 29:785–91. 10.3109/02688697.2015.1071320 26313119

[27] Moret J Cognard C Weill A Castaings L Rey A. The “Remodelling Technique” in the treatment of wide neck intracranial aneurysms. angiographic results and clinical follow-up in 56 cases. Interv Neuroradiol. (1997) 3:21–35. 10.1177/159101999700300103 20678369

[28] Wang F Chen X Wang Y Bai P Wang HZ Sun T. Stent-assisted coiling and balloon-assisted coiling in the management of intracranial aneurysms: a systematic review & meta-analysis. J Neurol Sci. (2016) 364:160–6. 10.1016/j.jns.2016.03.041 27084238

[29] Li MH Li YD Tan HQ Luo QY Cheng YS. Treatment of distal internal carotid artery aneurysm with the willis covered stent: a prospective pilot study. Radiology. (2009) 253:470–7. 10.1148/radiol.2532090037 19789235

[30] Becker GJ Benenati JF Zemel G Sallee DS Suarez CA Roeren TK. Percutaneous placement of a balloon-expandable intraluminal graft for life-threatening subclavian arterial hemorrhage. J Vascular Interventional Radiol. (1991) 2:225–9. 10.1016/S1051-0443(91)72286-0 1799760

[31] Marotta TR Buller C Taylor D Morris C Zwimpfer T. Autologous vein-covered stent repair of a cervical internal carotid artery pseudoaneurysm: technical case report. Neurosurgery. (1998) 42:408–12; discussion 12–3. 10.1097/00006123-199802000-00138 9482197

[32] Celil G Engin D Orhan G Barbaros C Hakan K Adil E. Intractable epistaxis related to cavernous carotid artery pseudoaneurysm: treatment of a case with covered stent. Auris Nasus Larynx. (2004) 31:275–8. 10.1016/j.anl.2004.03.007 15364363

[33] Yi AC Palmer E Luh GY Jacobson JP Smith DC. Endovascular treatment of carotid and vertebral pseudoaneurysms with covered stents. AJNR Am J Neuroradiol. (2008) 29:983–7. 10.3174/ajnr.A0946 18296552 PMC8128593

[34] Chen G Li J Xu G Qin S Gong J Yang M. Diagnosis and treatment of traumatic internal carotid artery pseudoaneurysm primarily manifested by repeated epistaxis. Turk Neurosurg. (2013) 23:716–20. 10.5137/1019-5149.JTN.7424-12.1 24310453

[35] Liu P Yang M Cai M Qin J Pan L. Treatment of pediatric traumatic intracranial pseudoaneurysm using endovascular covered stent: three case reports. World Neurosurg. (2016) 88:693 e1–6. 10.1016/j.wneu.2015.12.037 26724623

