# Supplementary figures and images for: Treatment of Traumatic Intracranial Pseudoaneurysms: A Single-Center Experience
Source: Front Neurol. 2021 Jun 25;12:690284. doi: 10.3389/fneur.2021.690284 (PMC8267006; doi:10.3389/fneur.2021.690284)

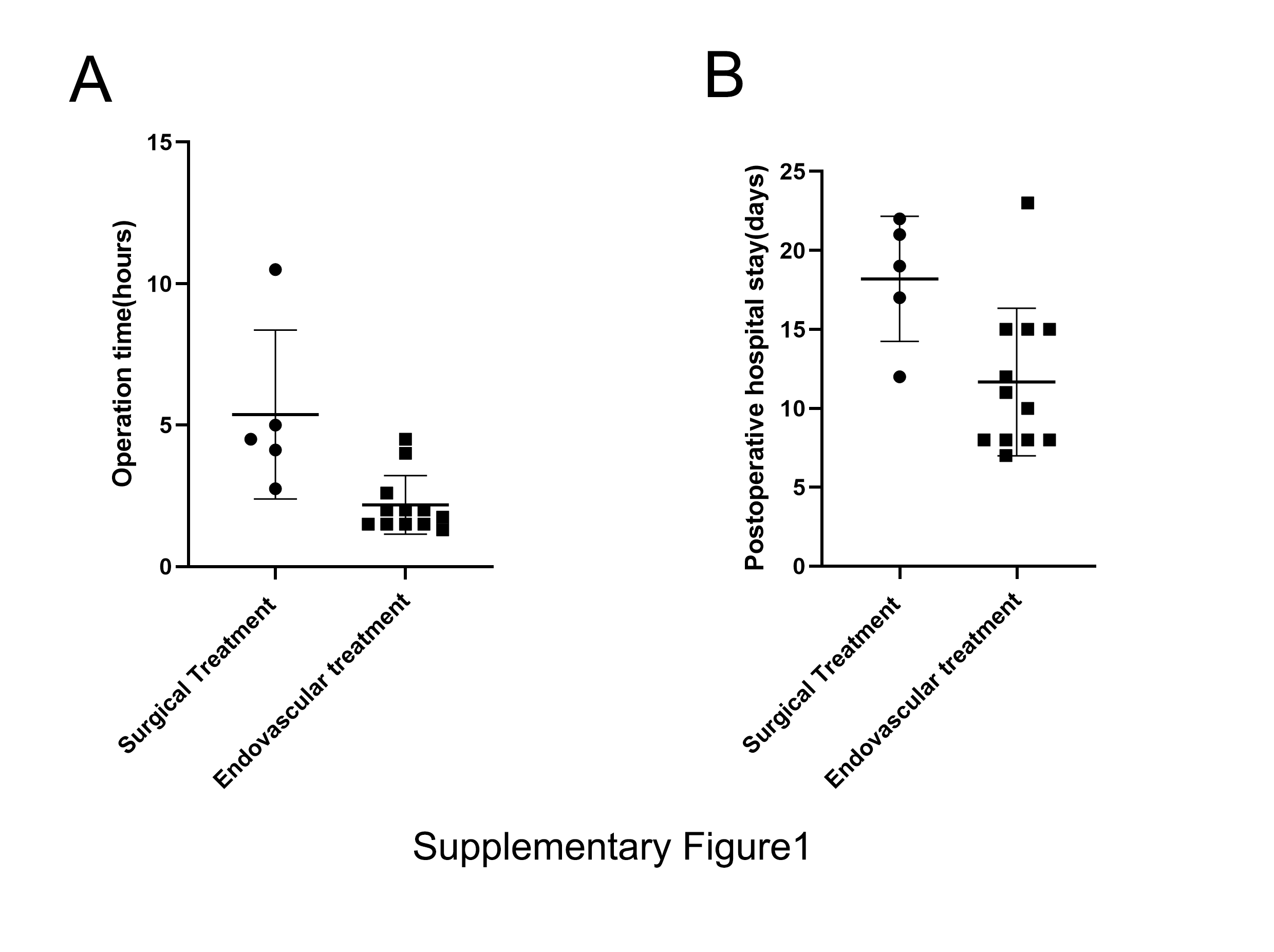

Supplement: Supplementary file 1 [file Image_1.TIF]
